# Suicide rates of migrants in United States immigration detention (2010–2020)

**DOI:** 10.3934/publichealth.2021031

**Published:** 2021-05-13

**Authors:** Parsa Erfani, Elizabeth T Chin, Caroline H Lee, Nishant Uppal, Katherine R Peeler

**Affiliations:** 1 Harvard Medical School, Boston, Massachusetts, USA; 2 Harvard T.H. Chan School of Public Health, Boston, Massachusetts, USA; 3 Department of Biomedical Data Science, Stanford University, Palo Alto, California, USA; 4 Harvard Business School, Boston, Massachusetts, USA; 5 Division of Medical Critical Care, Boston Children's Hospital, Boston, Massachusetts, USA

**Keywords:** migrant, immigrant, detention, incarceration, mental health, suicide

## Abstract

**Objective:**

We determined the annual suicide rate of migrants detained by U.S. Immigration and Customs Enforcement (ICE) in the past decade.

**Methods:**

We performed a retrospective cohort analysis of the annual suicide rates for ICE detainees from federal fiscal years (FY) 2010–2020. Death date and cause of death were directly extracted from publicly available ICE Freedom of Information Act (FOIA) Library, ICE death reports, and ICE press releases. Annual suicide rates were calculated as suicides per 100,000 person-years and suicides per 100,000 admissions.

**Results:**

From 2010–2019, the mean number of suicides per 100,000 person-years was 3.3 (standard deviation (SD): 2.6). In 2020, the suicide rate increased 5.3 times the prior 10-year average to 17.4 suicides per 100,000 person-years. When calculating suicide rate based on admissions per FY, the mean number of suicides from 2010–2019 per 100,000 admissions was 0.3 (SD: 0.3). In 2020, the suicide rate increased 11.0 times the prior 10-year average to 3.4 suicides per 100,000 admissions.

**Conclusion:**

In 2020, the detainee suicide rate increased substantially compared to the past decade. This may point to a worsening mental health crisis in ICE detention.

## 1. Introduction

Mental health care for migrants in U.S. Immigration and Customs Enforcement (ICE) detention has historically been substandard [1],[2]. Given that at baseline, ICE detainees suffer from higher rates of anxiety, depression, and post-traumatic stress disorder (PTSD), they are especially susceptible to stressors [3]. Time spent in immigration detention is a particular post-migration stressor that may exacerbate underlying mental health conditions [4]. Widespread failures to provide necessary mental health care to detainees and critical medical staff shortages put ICE detainees at an increased risk for suicide [5].

A recent study by Terp et al. reported that between 2018 and 2020, the proportion of deaths in ICE detention attributed to suicide approximately doubled since cause of deaths were last described in 2015 [6]. The annual suicide rates, however, have not been previously reported for migrants held in ICE detention. Here, we determine the annual suicide rates of ICE detainees over the past decade.

## 2. Materials and methods

We performed a retrospective cohort analysis of the death and suicide rates for ICE detainees from federal fiscal years (FY: October 1–September 30) 2010 to 2020. Data were obtained from the publicly available ICE Freedom of Information Act (FOIA) Library, ICE death reports, and ICE press releases [7]–[9]. Death date and cause of death were directly extracted. The average daily population (ADP), average length of stay, and total admissions per FY were obtained from public ICE sources [10],[11]. The number of non-COVID-19 related deaths and suicide deaths per FY were calculated. All years henceforth refer to FY.

Annual suicide rates were calculated as suicides per 100,000 person-years and suicides per 100,000 admissions. Suicides per 100,000 person-years were calculated as the number of suicides per year divided by the average daily population (ADP), with the resulting quotient multiplied by 100,000. The ADP is equal to the product of the annual number of admissions and mean length of stay and is used as the denominator for mortality rates to accommodate the high turnover and daily fluctuation in detainee populations. Corresponding annual death rates were also calculated.

## 3. Results

From 2010–2019, the mean number of deaths per FY in ICE detention was 8.9 (standard deviation [SD]: 1.7), 1.2 (SD: 0.9) of which were from suicide (13%). In 2020, the number of non-COVID-19 attributed deaths was 13, six of which were from suicide (46%) (Supplementary Appendix). Two of the six suicide deaths occurred during the COVID-19 pandemic, which lasted seven months of FY 2020 (March–September 2020).

**Figure 1.**
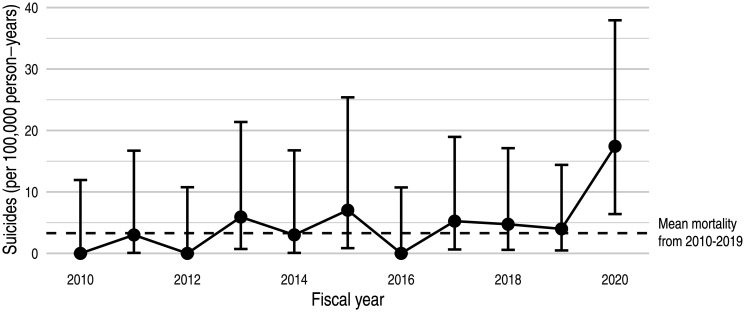
Suicide rate per 100,000 person-years in U.S. Immigration Detention Centers (FY 2010–2020). Point estimates (circles) and 95% confidence intervals (vertical bars) of trends in mortality for deaths attributed to suicide.

From 2010–2019, the mean number of suicides per 100,000 person-years was 3.3 (SD: 2.6). In 2020, the suicide rate increased 5.3 times the prior 10-year average to 17.4 suicides per 100,000 person-years (Figure 1). When calculating suicide rate based on admissions per FY, the mean number of suicides from 2010–2019 per 100,000 admissions was 0.3 (SD: 0.3). In 2020, the suicide rate increased 11.0 times the prior 10-year average to 3.4 suicides per 100,000 admissions.

## 4. Discussion and conclusions

The ICE detainee suicide rate was substantially higher in 2020 than the prior 10-year average. While Terp et al. point to an increased proportion of suicide deaths between 2018–2020 for ICE detainees, we find that when compared to the prior decade, the increased proportion and rate of suicide deaths occurred primarily in 2020 [6]. This may represent a worsening mental health crisis in ICE detention in the past year.

The increased suicide rate in 2020 may be related to increasing lapses in mental health care in ICE detention. A 2020 Congressional investigation revealed major issues in mental health care inside detention centers including delayed psychiatric appointments, placement of patients with mental health disease in solitary confinement, and falsified observation logs for suicidal patients [5]. Such gaps may result from chronic staffing shortages, as vacancy rates of 37–50% for psychiatrists and social workers have been previously reported in immigration detention [1]. Given the high prevalence of mental health disorders and stressors inside detention, these lapses are particularly harmful for this vulnerable population [3],[4].

An elevated suicide rate in 2020 may also be partially related to the increased mental health stressors on detainees during the COVID-19 pandemic. The number of suicides during the seven months of the pandemic (n = 2) in 2020 was greater than the average number of suicides per FY from 2010–2019 (mean: 1.2). During the COVID-19 pandemic, strict movement restrictions (such as use of 23.5 hour per day lockdowns and use of solitary confinement for medical isolation) were implemented to mitigate COVID-19 spread [12]. These measures that further limited freedom and social interactions, within an environment with limited opportunities for self-protection against COVID-19, may have exacerbated underlying mental health illness among migrants. The shifting of health care resources toward COVID-19 management may have also widened the existing mental health care gaps, leaving detainees at increased risk for suicide. Furthermore, increased length of detention has been associated with higher levels of anxiety, depression and PTSD among ICE detainees [3]. Given that the average length of stay in detention nearly doubled in 2020 (from 34.3 days in 2019 to 62.7 days in 2020), longer detention periods may have also increased the risk for suicide among detainees (Supplementary Appendix).

This study has several limitations. First, ICE's death data for the past decade were extracted from several sources. Death data from October 2018 to September 2020 were extracted from ICE's official public reporting of deaths, as required by the 2018 Department of Homeland Security Appropriations Bill [7]. Death data from October 2010 to May 2017 were extracted from ICE's FOIA library and death data from June 2017 to September 2018 were extracted from the American Immigration Lawyer Association (AILA) “Deaths at Adult Detention Centers” database [8],[9]. Deaths and their causes reported by the AILA were confirmed by corresponding ICE press releases [9]. Second, suicide is a rare event and given the small sample size, results should be interpreted with caution. The extrapolation of these results to future years is unknown. Third, immigration detention is a highly controlled and populated environment where detainees are closely monitored by facility staff and commonly share a sleeping area with more than 30 people [12]. Given the difficulty of committing suicide in such controlled settings, reported suicide rates likely grossly underestimate the rates of suicide attempts and suicidal ideation among detainees.

Our findings suggest that in 2020, in addition to the COVID-19 pandemic, there was a substantial increase in suicides inside ICE detention. Based on these data, we recommend the U.S. Department of Homeland Security pursue an independent investigation of suicides in 2020 to evaluate deficiencies in safety and mental health care services in detention and swiftly implement necessary changes to mitigate suicide risk, such as increasing mental health staffing, ensuring consistent checks for individuals on suicide watch, and ending use of solitary confinement. Given the inherent mental and physical health risks of detention, we also recommend the U.S. Congress limit immigration detention in line with international law and human rights standards [12].
