# Supplementary material for: Suicide rates of migrants in United States immigration detention (2010–2020)
Source: AIMS Public Health. 2021 May 13;8(3):416–20. doi: 10.3934/publichealth.2021031 (PMC8334629; doi:10.3934/publichealth.2021031)
Supplement: Supplementary file 1 [file publichealth-08-03-031-s001.pdf]

AIMS Public Health

AIMS Public Health, 8(3): 416–420.
DOI: 10.3934/publichealth.2021031
Received: 27 April 2021
Accepted: 11 May 2021
Published: 13 May 2021

http://www.aimspress.com/journal/aimsph

Brief report

Supplementary Tables and Figures

Analysis: https://github.com/etchin/detaineeSuicideAnalysis

Supplementary Table 1. Death and suicide rates in U.S. Immigration Detention Centers (FY 2010–2020).

Fiscal Year	Total Detained Population	Average Daily Population	Average Length of Stay	Deaths*	Death Rate per 100K Admissions*	Death Rate per 100K Person-Years*	Suicides (% Total Deaths*)	Suicide Rate per 100K Admissions	Suicide Rate per 100K Person-Years
2010	363,064	30,885	31.5	8	2.2	25.9	0 (0%)	0.0	0.0
2011	429,247	33,330	29.2	10	2.3	30.0	1 (10%)	0.2	3.0
2012	477,523	34,260	26.6	8	1.7	23.4	0 (0%)	0.0	0.0
2013	440,557	33,788	28.7	9	2.0	26.6	2 (22%)	0.5	5.9
2014	425,728	33,227	29.6	6	1.4	18.1	1 (17%)	0.2	3.0
2015	307,342	28,449	34.6	8	2.6	28.1	2 (25%)	0.7	7.0
2016	352,882	34,376	34.9	10	2.8	29.1	0 (0%)	0.0	0.0
2017	323,591	38,106	43.7	12	3.7	31.5	2 (17%)	0.6	5.2
2018	396,448	42,188	39.4	10	2.5	23.7	2 (20%)	0.5	4.7
2019	510,854	50,165	34.3	8	1.6	15.9	2 (25%)	0.4	4.0
2020	177,391	34,427	62.7	13**	7.3	37.8	6 (46%)	3.4	17.4

Note: *Excluding COVID-19 deaths; **An additional 8 deaths related to COVID-19 were reported in detention in FY 2020 (total deaths = 21).

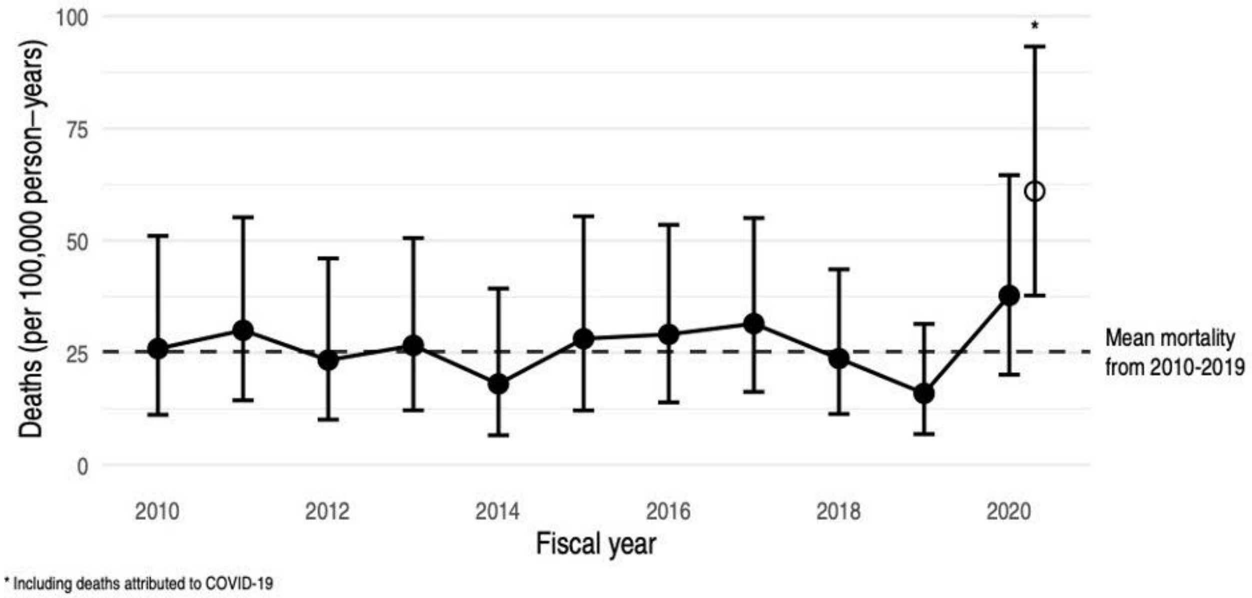

Supplementary Figure 1. Death rate per 100,000 person-years in U.S. Immigration Detention Centers (FY 2010–2020). Point estimates (circles) and 95% confidence intervals (vertical bars) of trends in mortality for deaths. For the 2020 FY, estimates for total non-COVID-19 attributed deaths and total deaths are referred to using solid and empty points, respectively.

AIMS Press

© 2021 the Author(s), licensee AIMS Press. This is an open access article distributed under the terms of the Creative Commons Attribution License (http://creativecommons.org/licenses/by/4.0)
